# Class I malocclusion with anterior crossbite and severe crowding

**DOI:** 10.1590/2176-9451.19.2.115-125.bbo

**Published:** 2014

**Authors:** Daltro Enéas Ritter

**Affiliations:** 1 Adjunct professor, Department of Orthodontics, Federal University of Santa Catarina (UFSC).Certified by the Brazilian Board of Orthodontics and Dentofacial Orthopedics (BBO).

**Keywords:** Severe discrepancy, Crossbite, Corrective Orthodontics

## Abstract

This article reports the orthodontic diagnosis and treatment planning carried out with a 14-year and 5-month-old female patient with esthetic and functional complaints. She presented an Angle Class I malocclusion, anterior crossbite and severe crowding in both maxillary and mandibular arches, in addition to a lightly concave straight facial profile. Orthodontic treatment did not require extraction. Crossbite was corrected by protrusion of upper teeth, which contributed to alignment and leveling of teeth, in addition to improving the patient's facial profile. The case was presented to the Brazilian Board of Orthodontics and Dentofacial Orthopedics (BBO) as a requirement for the BBO certification.

## Abstract (pt)

Esse artigo relata o diagnóstico, planejamento e execução do tratamento ortodôntico de uma paciente com 14 anos e 5 meses de idade, cuja queixa principal era estética e funcional. A paciente portava má oclusão de Classe I de Angle, mordida cruzada anterior e falta de espaço severo nas arcadas superior e inferior. O perfil facial era reto, com tendência a côncavo. O tratamento ortodôntico foi realizado sem necessidade de exodontias, com a correção da mordida cruzada por meio da projeção dos dentes superiores, o que auxiliou no alinhamento e nivelamento dentário, além de melhorar o perfil facial da paciente. Esse caso foi apresentado ao Board Brasileiro de Ortodontia e Ortopedia Facial (BBO) como parte dos requisitos para obtenção do título de Diplomado pelo BBO.

## INTRODUCTION

A 14-year and 5-month-old patient, in good health, with controlled allergic rhinitis, showed up for her first appointment. Her mother reported that the patient fell when she was eight years old, and fractured the incisal edge of tooth #41. At that point, the tooth was partially restored and remained as so with neither apical radiolucency nor sensibility until her first orthodontic appointment. The patient avoided smiling and showing her teeth while talking. Her major complaints comprised lack of space in both maxillary and mandibular arches and anterior crossbite. The patient reported the following: "*I am embarrassed of smiling. I want to have my teeth fixed because they are not aligned, which makes it difficult to bite*." The patient was in the descending pubertal growth spurt curve, 24 months after menarche. Her dental history included good oral hygiene, unchanged tongue position during physiologic movements and no orthodontic treatment.

## DIAGNOSIS

**Figure 1:**
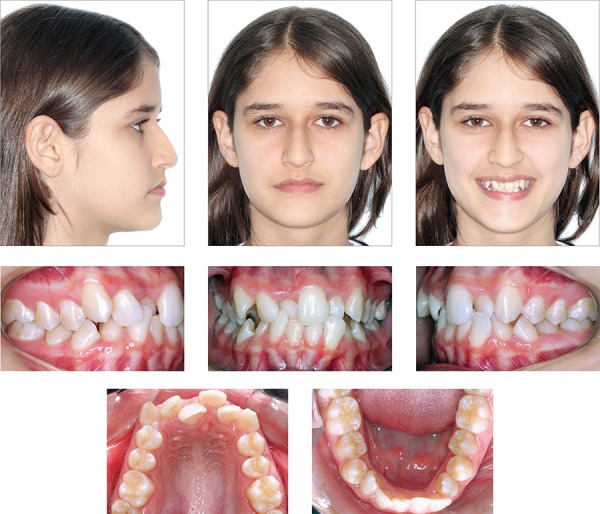
Initial facial and intraoral photographs.

The patient was an adolescent in the residual growth phase.^1^ She presented an asymmetrical face, with proportional facial thirds and spontaneous lip seal (Fig 1). She avoided smiling and showing her teeth, which hindered the assessment of spontaneous smile and the amount of tooth exposure at smile. Her forced smile revealed that her upper lip covered the gingival margin of her upper incisors.

The patient had a straight facial profile, with thin, retracted lips in relation to the Nasolabial line (S-UL = -1.0 mm and S-LL = 0 mm).^2^

**Figure 2:**
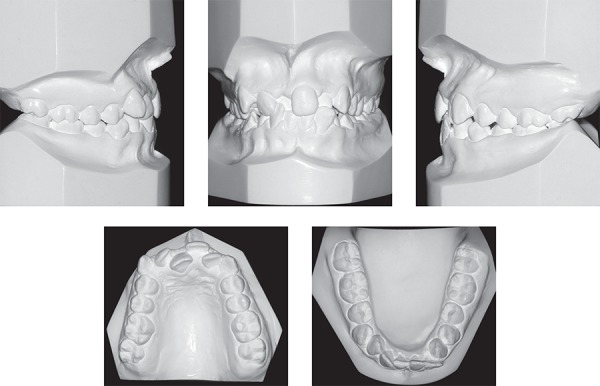
Initial casts.

From a dental point of view, she presented Angle Class I malocclusion (Figs 1 and 2), although her upper and lower canines were in end-to-end anteroposterior relationship. Additionally, she presented severe crowding in the maxillary and mandibular arches (maxillary discrepancy of -10 mm and mandibular discrepancy of -6 mm), and anterior crossbite including #11 and #22. Overjet between #21 and #31 was +3 mm, and -3 mm between #11 and #41. Tooth #41 was fractured and partially restored, given that restoration could not be properly carried out due to lack of space resulting from the malocclusion. Oral hygiene was good.

**Figure 3:**
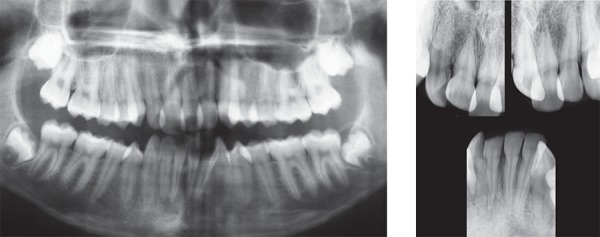
Initial panoramic and periapical radiographs of incisors.

Panoramic and periapical radiographs (Fig 3) revealed good root formation for all teeth, absence of apical radiolucencies around tooth #41 as well as absence of bone or dental anomalies. She had unerupted third molars at Nolla's stage 6 (initial root formation).

**Figure 4:**
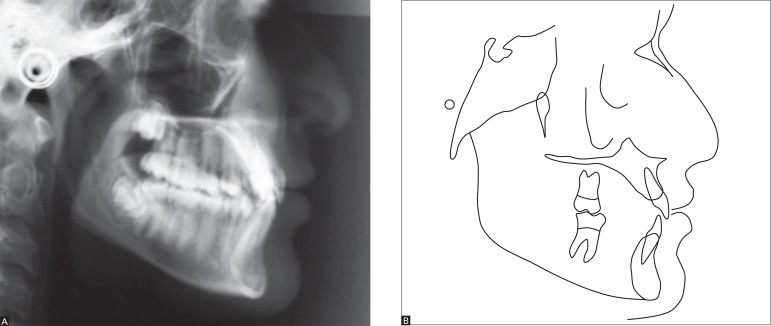
Initial (**A**) lateral cephalogram and (**B**) cephalometric tracing.

Cephalometric cephalograms and tracings (Fig 4) revealed a balanced skeletal pattern in the anteroposterior direction between the maxilla, the mandible and other facial structures (ANB= 1º and Angle of Convexity = -0.5º), with a predominantly horizontal growth pattern and mandibular plane (SN-GoGn = 25º, FMA = 20º and Y Axis = 54º). Upper and lower incisors were retroclined (1-NA = 20º, 1-NA = 6 mm, 1-NB = 17º, 1-NB = 4 mm and IMPA = 83.5º).The aforementioned cephalometric data are shown in Table 1.

**Table 1:** Cephalometric measurements.

	Measurements		Normal	A	B	Dif. A/B
Skeletal pattern	SNA	(Steiner)	82°	87°	87°	0
	SNB	(Steiner)	80°	86°	88°	2
	ANB	(Steiner)	2°	1°	-1°	2
	Angle of convexity	(Downs)	0°	-0.5°	-4.5°	4
	Axis Y	(Downs)	59°	54°	53°	1
	Facial angle	(Downs)	87°	94.5°	97°	2.5
	SN-GoGn	(Steiner)	32°	25°	23°	2
	FMA	(Tweed)	25°	20°	17.5°	2.5
Dental pattern	IMPA	(Tweed)	90°	83.5°	92°	8.5
	1.NA	(Steiner)	22°	20°	36°	16
	1-NA	(Steiner)	4 mm	6 mm	10 mm	4
	1.NB	(Steiner)	25°	17°	25°	8
	1-NB	(Steiner)	4 mm	4 mm	6 mm	2
	1.1 – Interincisal angle	(Downs)	130°	142°	120°	22
	1-APo	(Ricketts)	1 mm	2.5 mm	5 mm	2.5
Profile	Upper lip – Line S (S-UL)	(Steiner)	0 mm	-1.0 mm	-1.0 mm	0
	Lower lip – Line S (LL-S)	(Steiner)	0 mm	0 mm	0 mm	0

## TREATMENT PLAN

The patient had a harmonious and proportional face (front view), but presented a straight profile, which is worrying for a 14-year-old, given that one's profile tends to become more concave with time.^3,4^ For this reason, treatment plan avoided extractions and included protrusion of retroclined incisors, as well as increasing lip support and volume, which resulted in a more convex profile.

The patient presented Angle Class I malocclusion, with anterior crossbite of teeth #11 and #22. Given that her central mandibular and maxillary incisors were retroclined, treatment plan aimed at obtaining mesio-distal space by means of a fixed orthodontic appliance, providing protrusion of anterior teeth in normal occlusion, increased arch circumference, and protrusion of maloccluded teeth in order to meet normal standards.^5^ Negative discrepancy was severe in both maxillary (-10 mm) and mandibular arches (-6 mm). Mandibular canines were visibly retroclined, with reduced distance between canines. Protrusion of maxillary and mandibular incisors was able to solve such negative discrepancy and increase the distance between canines and molars in both maxillary and mandibular arches, particularly in the mandibular canines.

An alternative treatment plan would include extraction of the four first premolars. This treatment option, however, does not allow enough protrusion of incisors and, as a result, does not improve lip support. Moreover, after some years, it would worsen the patient's facial profile.^3,4,6,7^

## TREATMENT PROGRESS

Edgewise 0.022 x 0.028-in orthodontic brackets were placed in the maxillary arch, except for teeth #11 and 22 (maloccluded). Treatment began with Twist-flex 0.015-in steel archwire placed for initial alignment and leveling. Subsequently, 0.012, 0.014, 0.016 and 0.018-in stainless steel archwires were progressively installed every 30 days, with omega loops mesially adjusted to the first molars. The omega loops were adjusted in 0.05 mm on each side on every orthodontic visit, increasing arch circumference and length, and, as a result, establishing mild and continuous protrusion of incisors with expansion of the arches.

**Figure 5:**
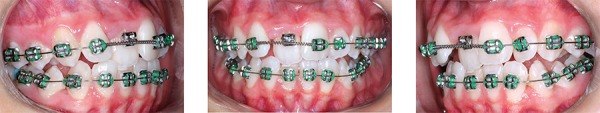
Intraoral photographs 8 months after treatment onset.

**Figure 6:**
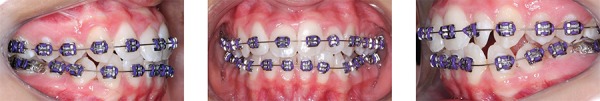
Intraoral photographs 12 months after treatment onset.

Once the 0.018-in steel wire had been installed, open springs were compressed between teeth #11 and 22 to create space between them. At this point, orthodontic appliance was installed on these teeth. Buccal traction of maloccluded teeth was performed with mild-force elastomeric chains between the 0.018" arch and the bonded appliances. At this stage, maxillary incisors were slightly protruded, thus providing enough space to correct the malocclusion (Fig 5). By the time the 0.020" stainless steel wire was installed, the incisors had been satisfactory protruded, thus providing enough mesiodistal space for buccal inclination of #11 and #22 (Fig 6).

After maloccluded teeth were corrected, the appliances of #11 e #22 were replaced and a new 0.014" stainless steel wire was installed for teeth alignment and leveling. Subsequently, 0.016, 0.018, 0.020-in and 0.019 x 0.025-in stainless steel wires were progressively installed for individual torque control and treatment finishing.

Edgewise 0.022 x 0.028-in orthodontic brackets were placed in all teeth of the maxillary arch. It is worth noting that on teeth #31, 33 and 43, the appliances were provisionally bonded in a more cervical direction so as to avoid occlusal contact with antagonist teeth. The occlusal bracket wings of teeth #35, 44 and 45 had to be partially worn after bonding due to occlusal interference. Initially, a 0.015-in Twist-flex steel alignment and leveling archwire was installed. Subsequently, 0.012, 0.014, 0.016-in and 0.018-in stainless steel archwires were progressively installed every 30 days, with omega loops mesially adjusted to the first molars. Similarly and concurrently to the maxillary arch, the omega loops were adjusted in 0.05 mm on each side, increasing arch circumference and length, and, as a result, establishing mild and continuous protrusion of incisors.

As protrusion of upper and lower incisors progressed, more space was created for rebonding of teeth #31, 33 and 43 which, as it has been previously mentioned, were initially bonded in a non-ideal position. These teeth were rebonded on an average of three to four times, until their ideal position (in comparison to the other teeth) could be reached. After the 0.019 x 0.025-in stainless steel rectangular archwire was installed, with it acting over the torques and establishing correct intercuspation, the appliance was removed.

Retention consisted of a wraparound removable appliance in the maxillary arch, used full-time (except for meals and oral hygiene) during six months, 12 hours per day during the following six months and while sleeping during the last six months of retention. After a retention period of a year and a half, the patient was advised to use the maxillary retainer two nights a week while sleeping for an indefinite period of time.

In the mandibular arch, a 0.020-in stainless steel wire intercanine bar was installed to ease incisors and canines interproximal space. This retainer was used for an indefinite period of time. Third molars were extracted six months before treatment completion.

## RESULTS

**Figure 7:**
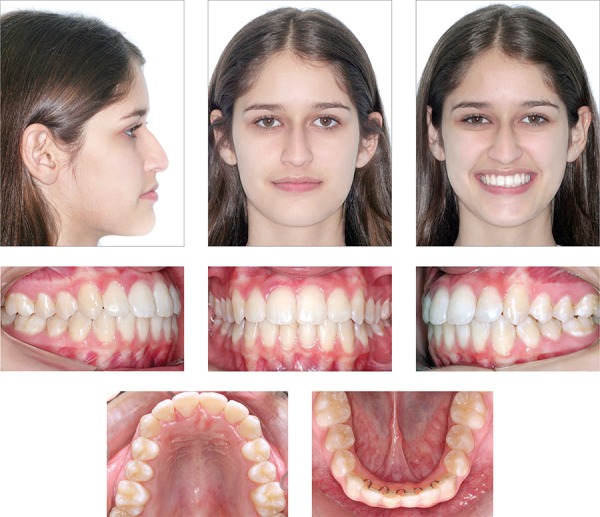
Initial facial and intraoral photographs.

At treatment completion, patient's self-esteem had significantly improved. Good facial proportions were observed in frontal view, with patient's profile improved due to an increase in lip volume as a result of incisor protrusion (Fig 7). Horizontal residual mandibular growth was greater than expected, given that menarche had occurred one year and a half before treatment onset. If incisor protrusion had not been planned, patient's profile would be clearly concave. Protrusion allowed patient's profile to favorably develop with age.^6,7^

**Figure 8:**
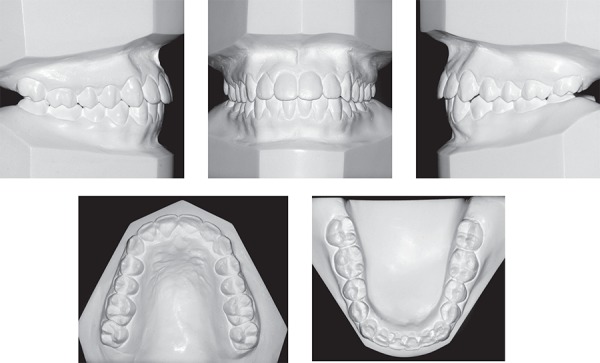
Final casts.

Molar and canine relationships were obtained in key to occlusion (Figs 7 and 8). Anterior crossbite and discrepancy of upper and lower models were corrected by protrusion of upper and lower incisors and mild expansion of the arches (Tab 2).The distance between lower molars increased in 2 mm, while the distance between upper molars increased in 3 mm during treatment. As for the distance between lower canines, it increased in 5 mm, whereas between upper canines, it increased in 1.5 mm. The greater increase in distance between lower canines was a result of accentuated lingual inclination of teeth #33 and 43, which was corrected during treatment.^8^ The average distance between lower canines in untreated patients is 25 mm, whereas in the case reported herein it was of 22 mm. Normal overjet and overbite were obtained with anterior disocclusion guides on the incisors, and lateral disocclusion guides on right and left canines.

**Table 2:** Measurements of transversal distances of the dental arches (mm).

Cast / phase measurements	A	B	Dif. A/B
Distance between lower canines	22 mm	27 mm	5 mm
Distance between lower molars	43 mm	45 mm	2 mm
Distance between upper canines	36 mm	37.5 mm	1.5 mm
Distance between upper molars	49 mm	52 mm	3 mm

**Figure 9:**
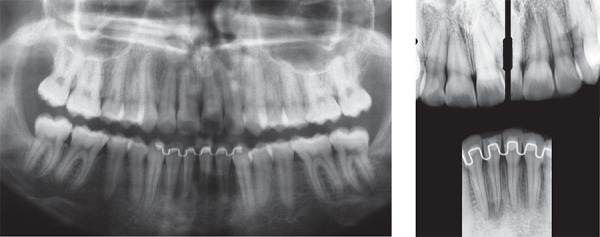
Final panoramic and periapical radiographs of incisors.

**Figure 10:**
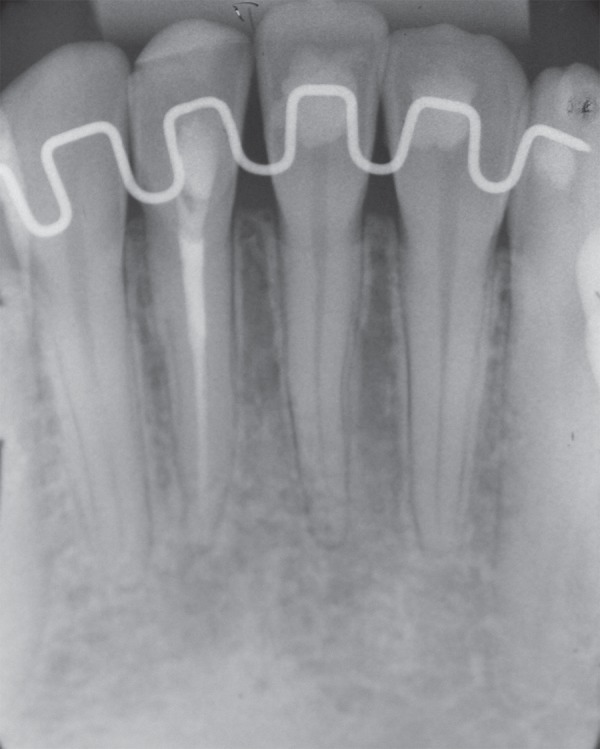
Control periapical radiograph of tooth #41 six months after endodontic treatment.

Final panoramic radiograph revealed root parallelism, whereas periapical radiographs revealed absence of root resorption (Fig 9). During treatment, restoration of tooth #41 was recommended. However, the dentist advised the patient to wait for treatment completion in order to have such procedure carried out. At treatment completion, tooth #41 presented apical radiolucencies, as revealed by the final periapical radiograph, and the patient was referred to a specialist for endodontic treatment and restoration of tooth #41, all of which were carried out one month after the orthodontic appliance had been removed. Radiographic control taken six months afterwards revealed periapical repair (Fig 10).

**Figure 11:**
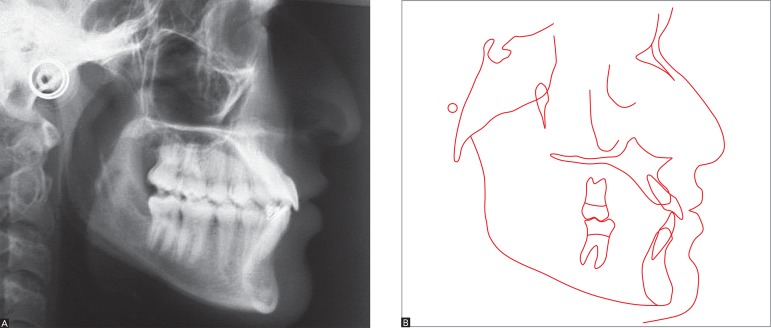
Final (**A**) lateral cephalogram and (**B**) cephalometric tracing.

Final cephalometric tracings and cephalogram (Fig 11, Tab 1) highlighted that, by the end of treatment, maxillary incisors were in increased protrusion (1-NA = 36º and 1-NA = 10 mm), although clinically appropriate, whereas mandibular incisors were well positioned (1-NB=25º, 1-NB=6 mm and IMPA=92º).

At treatment completion, the mandible was more anteriorly positioned in relation to the maxilla, with ANB = -1º and angle of convexity = 0.5º, both clinically acceptable. The mandibular plane revealed mild anticlockwise movement, observed by a reduction in the mandibular plane angles (SN-GoGn and FMA) and Y Axis.

**Figure 12:**
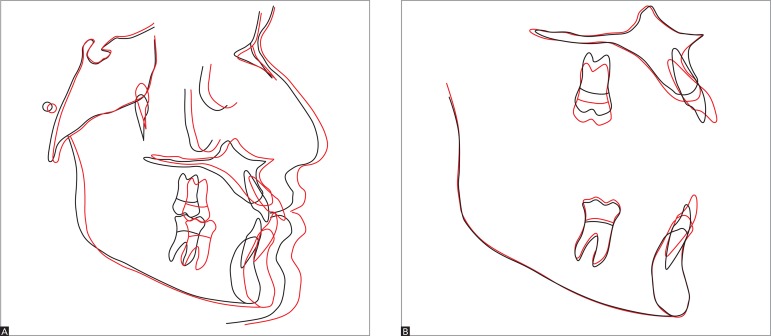
Initial (**black**) and final (**red**) cephalometric tracing total (**A**) and partial (**B**) superimposition.

Cephalometric tracing superimposition revealed that, during treatment, mandibular growth in the horizontal direction of the maxilla was greater (Fig 12), which could be explained by the reduced anterior movement of point A in relation to point B, showing little maxillary growth in relation to the maxilla as a result of the buccal inclination of maxillary incisors. An increase in the inclination of maxillary incisors, with palatal root movement as a consequence, may have been influenced by the posterior positioning of point A, giving the false impression of insufficient maxillary growth.

## FINAL CONSIDERATIONS

The results were obtained as planned: excellent facial esthetics, anterior teeth in normal occlusion and improvements in alignment and leveling. The patient demonstrated to be satisfied with the results, which improved her self-esteem.

In spite of the anterior displacement of the mandible being greater than expected, patient's profile remained straight as a result of protrusion of incisors, which provided lip support and volume with excellent esthetics. Key to occlusion was obtained for molars and canines with ideal occlusion guides.
